# Soil viruses: Understudied agents of soil ecology

**DOI:** 10.1111/1462-2920.16258

**Published:** 2022-11-05

**Authors:** Janet K. Jansson

**Affiliations:** ^1^ Department of Biosciences, Earth and Biological Sciences Directorate Pacific Northwest National Laboratory Richland Washington USA

Over the past couple decades, there has been intense exploration of the soil microbiome using modern sequencing and bioinformatics approaches. These studies have revealed a vast diversity of microorganisms across a variety of habitats (Thompson et al., 2017) and how differences in the environment, for example, with climate change, influence the soil microbial community composition (Jansson & Hofmockel, 2020). The focus of these studies has primarily been on microbial cellular organisms, including bacteria, archaea and fungi. In contrast, in depth study of soil viruses has been largely neglected, until recently. There are several reasons that soil viruses are less studied, including difficulties with extraction of viruses from soil particles, difficulties with classification of soil viruses based on microscopic imaging, and reliance on the small proportion of microbial hosts that can be cultivated. Now that comprehensive soil metagenomes are available the current trend has been to survey the metagenomes for viral sequences. This approach has revealed that soil viruses are incredibly abundant, highly diverse, and largely uncharacterized (Paez‐Espino et al., 2016). This so‐called ‘*viral dark matter*’ presents an incredible research opportunity for the future. Here, I will highlight some of these potential research avenues based on what is known to date.

## SOIL VIRUSES ARE HIGHLY ABUNDANT

Current counts of soil viral abundances have revealed that they are as abundant, or more abundant, than their hosts. Most information about viral numbers in soil has been obtained by counting of bacterial viruses (bacteriophage) that can be identified by microscopy and/or cultivated with their bacterial hosts (Williamson et al., 2013). Direct microscopic counts of virus‐like particles (VLPs) from different soil types revealed approximately 10^8^–10^10^ VLP per gram dry weight of soil (Williamson et al., 2017), with higher numbers in forest soils when compared to agricultural soils (Williamson et al., 2005). However, the true number of soil viruses may be even higher than that obtained by microscopy, because many viruses are intracellular and not able to be imaged separately from their hosts. Even free viruses are often difficult to distinguish from the background of soil particles and the subcellular size of many viruses has further impeded their direct visualization in soil. However, DNA viruses range greatly in size from 20 nm to giant viruses that are up to 500 nm in diameter. The size and shape of viruses largely depend on the size of their genomes and protein arrangements that surround the genome. They are normally 20–50 times smaller than bacterial cells (Kuzyakov & Mason‐Jones, 2018), but some giant viruses recovered from permafrost soil are larger than typical bacteria (Legendre et al., 2014).

The advent of metagenomics issued in a new opportunity to scan different habitats for viral sequences (Edwards & Rohwer, 2005). Sequencing overcame the limitation with reliance on cultivation and/or microscopy for detection of viruses. With increases in sequencing depth, there was the possibility to get increasingly better coverage of DNA viral sequences. Currently, several complete genomes of novel soil viruses have been obtained from soil metagenomes (Wu, Davison, Nelson, et al., 2021). Extraction of viral particles prior to metagenome sequencing has been shown to increase recovery of viral populations over bulk sequencing approaches (Santos‐Medellin et al., 2021).

Although we know more about soil DNA viruses based on traditional microscopic and culture‐based analyses, RNA viruses are also abundant in soil. Recent screenings of soil RNA sequences (metatranscriptomes) have revealed a diversity of RNA viruses in different grassland soils (Starr et al., 2019; Wu, Davison, Gao, et al., 2021). Many of the detected RNA viruses have bacterial hosts, but several are predicted to have eukaryotic hosts. Although there are too few studies to make sweeping generalizations, there is a trend towards different dominant RNA viruses in different soil habitats. For example, different RNA viruses had higher representation in different grassland soils: M*itoviridae* from a CA annual grassland soil (Starr et al., 2019) and *Reoviridae* in Kansas native prairie soil (Wu, Davison, Gao, et al., 2021).

There are still several remaining research questions to be addressed. These include, but are not limited to the following: What are the hosts of soil viruses? The vast majority have not yet been linked to their hosts. Are the soil viruses that have been detected by sequencing approaches active, inactive or dead? Recently, stable isotopes were used to distinguish active from inactive viruses in a peat soil (Trubl et al., 2021). This approach shows great promise for application to other soil ecosystems. Another question is whether specific soil bacteriophage lysogenic or lytic and what environmental changes trigger transitions between viral lifestyles?

## SOIL VIRUSES HAVE AUXILIARY METABOLIC GENES

Studies of soil viral sequences in soil metagenomes have shown that some viral genomes contain auxiliary metabolic genes (AMGs) that are not required for normal viral replication and reproduction. For example, a viral gene that encoded an endomannanase enzyme was detected in permafrost metagenomes and functionally validated (Emerson et al., 2018). Recently, another AMG that encoded a chitosanase enzyme was not only functionally characterized but also crystalized to obtain the protein structure (Wu et al., 2022). The protein structure was used to predict the mode of action of the viral chitosanase. Interestingly, the protein was comprised of two domains: one was typical of some endoglucanase enzymes, whereas the other was a novel, loopy domain. The viral chitosanases were phylogenetically distinct from chitosanases in bacteria and fungi. In addition, soil viral chitosanases grouped separately from those in other ecosystems, such as marine systems. The implication of these findings is that soil viruses have the potential to provide metabolic reactions that can complicate those of their hosts. Many other potential AMGs have been detected in soil viral sequences from soil metagenomes. These range in potential function, including nutrient cycling of carbon, nitrogen compounds, lipid and protein metabolism and host metabolism and energy generation (Wu, Davison, Nelson, et al., 2021).

There are several remaining questions to address, including the following: What are the functional roles of different kinds of proteins that are expressed from AMGs carried on soil viruses? Does AMG expression benefit survival of the host under specific environmental conditions?

## SOIL VIRUSES PLAY A PREVIOUSLY UNRECOGNIZED ROLE IN SOIL ECOLOGY

Several recent studies have shown that soil viruses are influenced by changes in their environment. For example, vOTUs were higher in native prairie compared with conventionally tilled soils (Cornell et al., 2021). Changes in climate can also result in shifts in viral lytic and lysogenic lifestyles (and climate change; Wu, Davison, Nelson, et al., 2021). These impacts on soil viruses can have cascading effects on their hosts and environment. For example, transition of a temperate phage to a lytic cycle results in killing of their hosts. Often, the most dominant bacteria are those that are lysed, leaving room for less abundant members of the soil microbiome to grow and take their place. This is the ‘kill the winner’ hypothesis (Våge et al., 2014). Alternatively, during lysogeny, viruses can replicate together with their hosts—a process that is higher with higher host densities, via the ‘piggyback the winner’ hypothesis (Knowles et al., 2016). As the soil environment is impacted by land management or climate change, soil viruses are also influencing the ability of their hosts to survive and or adapt. When the hosts are lysed, they release carbon and nutrients into the soil environment that are subsequently consumed by other members of the soil biota. This sidestepping of the soil food web, whereby bacteria are consumed by protists, or other predators, is known as the ‘viral shunt’. Ultimately, the recycling of soil nutrients by heterotrophs can impact soil ecology by their entrapment in microbial bodies. As they die, the resulting necromass can serve to store the soil carbon and may be a valuable carbon sink if entombed in soil nanopores (Kuzyakov & Mason‐Jones, 2018)—particularly if associated with deep rooting perennial grasses that can drive the carbon deeper into the soil. This aspect of soil viral ecology could be important for helping to store carbon in deep soils but needs to be further explored to validate its potential.

Another way that soil viruses can contribute to soil ecology is through their expression of AMGs, many of which are predicted to play a role in cycling of carbon and other nutrients. For example, the chitosanase AMG described above expresses a functional chitosanase enzyme. Therefore, it could play a key role in decomposition of chitin that is an abundant carbon polymer in many soils as a product of decomposition of fungal cell walls and insect exoskeletons. In this example, the chitosanase enzyme was predicted to reside on a proteobacterial phage from a forest soil (Wu et al., 2022). Thus, it is intriguing to hypothesize that the viral chitosanase enzyme (V‐Csn) contributes towards chitin metabolism by its bacterial host to aid in nutrient acquisition by its host.

Remaining questions to address include the following: Does the viral shunt aid in soil carbon storage? What other types of AMGs are carried on soil viruses, including those that potentially generate energy and trigger dormancy in their hosts? Do different types of bacteriophage primarily express AMGs during lysogenic or lytic cycles, or both?

## CONCLUSIONS

In summary, recent explorations of soil viruses are beginning to unveil not only their identities but also their functional roles in the soil environments. However, there remains much to be learned about how different soil viruses interact with their hosts and how different environmental conditions influence their interactions. Today, most of the soil viruses remain uncharacterized except for their sequence similarities to known viruses. However, the majority of soil viruses are novel and not similar to known viruses. In addition, there have been few studies of isolated soil viruses that are interacting with their hosts, other than well‐characterized model viruses that have been easy to cultivate. Because soil viruses are so abundant and so responsive to changes in their environment, the downstream implications on their hosts and on the soil ecosystem can be profound. Therefore, the study of soil viruses and their influence on soil ecology represents a tremendous future research opportunity.

## AUTHOR CONTRIBUTION

J.K.J wrote the manuscript.

## CONFLICT OF INTEREST

J.K.J. has no conflicts of interest.

## Data Availability

No data are submitted with this review.
